# Machine learning identifies prognostic subtypes of the tumor microenvironment of NSCLC

**DOI:** 10.1038/s41598-024-64977-7

**Published:** 2024-07-01

**Authors:** Duo Yu, Michael J. Kane, Eugene J. Koay, Ignacio I. Wistuba, Brian P. Hobbs

**Affiliations:** 1 Division of Biostatistics, Institute for Health & Equity, Medical College of Wisconsin, Milwaukee, WI USA; 2 Department of Biostatistics, Yale School of Public Health, New Haven, CT USA; 3 Department Radiation Oncology, The University of Texas MD Anderson Cancer Center, Houston, TX USA; 4 Department of Translational Molecular Pathology, The University of Texas MD Anderson Cancer Center, Houston, TX USA; 5 Department of Population Health, Dell Medical School, The University of Texas at Austin, 1601 Trinity St., Austin, TX 78712 USA

**Keywords:** Precision oncology, Thoracic tumor, Biomarkers, Cancer immunity, Survival prediction, Lung cancer, Immunology, Biomarkers

## Abstract

The tumor microenvironment (TME) plays a fundamental role in tumorigenesis, tumor progression, and anti-cancer immunity potential of emerging cancer therapeutics. Understanding inter-patient TME heterogeneity, however, remains a challenge to efficient drug development. This article applies recent advances in machine learning (ML) for survival analysis to a retrospective study of NSCLC patients who received definitive surgical resection and immune pathology following surgery. ML methods are compared for their effectiveness in identifying prognostic subtypes. Six survival models, including Cox regression and five survival machine learning methods, were calibrated and applied to predict survival for NSCLC patients based on PD-L1 expression, CD3 expression, and ten baseline patient characteristics. Prognostic subregions of the biomarker space are delineated for each method using synthetic patient data augmentation and compared between models for overall survival concordance. A total of 423 NSCLC patients (46% female; median age [inter quantile range]: 67 [60–73]) treated with definite surgical resection were included in the study. And 219 (52%) patients experienced events during the observation period consisting of a maximum follow-up of 10 years and median follow up 78 months. The random survival forest (RSF) achieved the highest predictive accuracy, with a C-index of 0.84. The resultant biomarker subtypes demonstrate that patients with high PD-L1 expression combined with low CD3 counts experience higher risk of death within five-years of surgical resection.

## Introduction

Non–Small-Cell Lung Cancer (NSCLC) is a common subtype of lung cancer that accounts for 76% of all lung cancer cases in the United States^1,2^, and 85% of all lung malignancies worldwide^3^. With the advent of targeted therapy and immunotherapies, especially FDA-approved therapies for stage IV EGFR-positive NSCLC, a rapid decline in mortality from NSCLC has been observed in recent decades^4,5^. Nevertheless, the survival rate of NSCLC remains low, with a 5-year survival rate of less than 25%^6^. Furthermore, more than 75% of NSCLC cases are diagnosed in advanced stages (IIIA-IV), which results in an even lower survival rate^7,8^. Precise characterization of prognosis is pivotal to optimal patient management.

In addition to the commonly known factors that are associated with prognosis, such as stage of the disease, age, and sex^9^, recent advances in immunology have revealed prognostic biomarkers that describe the tumor microenvironment (TME)^10^. Programmed Death-Ligand 1 (PD-L1) has been recognized as a type of immunosuppressive checkpoint protein on tumor cell^11,12^. The binding of PD-L1 to programmed cell death 1 (PD-1) reduces the proliferation of CD8^+^ and CD4^+^ cells and induces apoptosis^13^. The antibody-mediated blockade of PD-L1 can result in durable tumor regression and can prolong stabilization of disease in patients with advanced cancers, including non–small-cell lung cancer (NSCLC), melanoma, and renal-cell cancer^12,14^. However, the prognostic role of PD-L1 expression in NSCLC remains contentious as conflicting results have been obtained from various studies. The variability in study results regarding PD-L1's prognostic role could be attributed to multiple factors, such as differences in subtypes (i.e., squamous cell carcinoma [SCC] and adenocarcinoma [AC])^15^, heterogeneity in clinical studies on NSCLC (where clinicopathological factors may vary), the use of diverse scoring methods, and different cutoff levels (details of which are discussed in the next paragraph)^16^. For instance, the association between PD-L1 expression and overall survival (OS) in AC patients has shown contradictory results. Some studies report that AC patients with high PD-L1 expression (defined at thresholds of 50% and 1%) experience shorter survival times^16–19^. Conversely, Cooper et al., using immunohistochemistry (Merck; clone 22C3) and considering a 50% expression threshold as indicative of high expression, found in a study of 678 stages I-III NSCLC patients that high PD-L1 expression appears to be a favorable prognostic factor in early-stage disease^20^.

Due in part to the absence of statistical methods for identifying biomarker thresholds from multivariable analysis adjusted for clinical prognosis, thresholds for PD-L1 positivity varied substantially in clinical trials developing immune checkpoint inhibitors (ICIs). For example, the KEYNOTE trials of pembrolizumab (Clinical- trials.gov: NCT01295827 and NCT01905657) scored patients for immune checkpoint expression by the presence of the PD-L1 protein on the surface of tumor cells (TC). These single-arm studies evaluated efficacy in relation to the proportion of tumor cells expressing PD-L1 with thresholds: 1%, 25%, 50%, 75%, etc. In addition to PD-L1, thresholds applied to multiple TME biomarkers have been proposed for prognostic subgroup classification^10,21^. Considering both expression levels of PD-L1 and specific tumor-infiltrating lymphocytes as candidate biomarkers to predict the immune response, Teng et al. proposed to classify cancers based on T-cell infiltration and PD-L1 into four different types of TMEs: tumor-infiltrating lymphocytes TIL^+^PD-L1^+^, TIL^+^PD-L1^−^, TIL^−^PD-L1^+^, TIL^−^PD-L1^−^^21^. Furthermore, the same rule has been employed to classify NSCLC patients (who are treated with definitive surgical resection) by % tumor PD-L1 expression (high vs. low) and density of TIL (high vs. low) via CD3 count into four immune-pathology groups: CD3^hi^PD-L1^hi^, CD3^hi^PD-L1^lo^, CD3^lo^PD-L1^hi^, and CD3^lo^PD-L1^lo^^10^. The study found that patients with immune-activated tumors (CD3^hi^PD-L1^lo^) had the highest overall survival rate at 5 years, while patients with immune-inhibited tumors (CD3^lo^PD-L1^hi^) experienced the worst 5-year survival.

In current practice, biomarker thresholds are defined by summary statistics of the observed data or domain experts. Moreover, the interdependence between biomarkers and known prognostic factors is often neglected in biomarker analysis. Neglecting this correlation may lead to the misattribution (to biomarkers) of trends in prognosis that are of clinical orientation. Few studies have developed statistical methods to estimate sub-region boundaries among multiple biomarkers. Guo et al.^22^ proposed SCUBA, a Subgroup ClUster-based Bayesian Adaptive design, to partition biomarker subspaces for biomarker-guided therapies. The authors make the limiting assumption that boundaries of the partitions are linear, which is unnecessary with recent advances in machine learning.

In this study, we demonstrate a data-driven approach to identify multi-dimensional biomarker thresholds based on regression and machine learning. Models are trained to predict overall survival for non-small cell lung cancer (NSCLC) who are treated with surgical resection on the basis of PD-L1 expression and CD3 count, and ten baseline patient characteristics. To generalize the biomarker inference beyond the observed sampled data points, while adjusting for correlation between biomarkers and clinical prognostic factors, a novel algorithm for data augmentation through synthetic patient profile simulation is applied. Thereafter, prognostic subtypes are delineated from subregions of the bivariate biomarker domain of PD-L1 and CD3.

## Results

### Statistical analysis

A total of 423 NSCLC patients who were treated with definitive surgical resection between December 2000 through February 2012 are included in the analysis. With a follow-up of 10 years, 219 patients died following surgical resection during the period of observation. Patient baseline characteristics are summarized in Table 1. To investigate prognostic subpopulation heterogeneity attributable to the tumor immune microenvironment, the CD3 cell counts and PD-L1 expression percentages were standardized. CD3 cell counts were directly normalized with Z-scores resulting in a normalized range of [− 1.90, 4.18]. For PD-L1 expression percentage, Z-score standardization was applied to the log-transformed values, resulting in a normalized range of [− 2.50, 2.21]. Based on univariate Cox proportional hazard regression, we observed higher CD3 count was significantly associated with a lower relative rate of death with a hazard ratio (HR) of 0.76 (P-value = 0.001), Table 1. Regression analysis, however, failed to identify a significant association between PD-L1 expression and survival [HR = 1.10 (P-value = 0.157)], Table 1.

**Table 1:** Characteristics of NSCLC patients.

Characteristics	Patient cohort (n = 423)	Hazard ratio of univariate Cox regression	P-values of univariate Cox regression
Median age at surgery in years (IQR)	67 (60–73)	1.03	< 0.001
Sex			
Female (%)	193 (46)	–	–
Male (%)	230 (54)	1.46	0.006
Race			
Caucasian (%)	370 (87)	–	–
Others (%)	53 (13)	0.86	0.488
Smoking status			
Current (%)	171 (40)	–	–
Former/never (%)	252 (60)	0.94	0.657
T status			
T1 (%)	153 (36)	–	–
T2 (%)	193 (46)	1.21	0.222
T3/4 (%)	77 (18)	2.15	≪ 0.001
N status			
N0 (%)	290 (69)	–	–
N1/2 (%)	133 (31)	2.25	≪ 0.001
Final patient stage			
I (%)	234 (55)	0.33	≪ 0.001
II (%)	118 (28)	0.63	0.012
≥ III (%)	71 (17)	–	–
Squamous (%)	161 (38)	1.70	≪ 0.001
Adjuvant therapy* (%)	143 (34)	1.12	0.411
Median lesion size in cm (IQR)	3.0 (2.2–4.5)	1.19	≪ 0.001
Median CD3 + cell count (IQR)	1560 (1052–2221)	0.79	0.001
Median percent tumor cell PD-L1 expression (IQR)	1.85 (0.74–7.57)	1.10	0.157

*The adjuvant therapy types were summarized in Supplemental Materials Table S1.

### Survival prediction

Motivated by prior studies, we hypothesized that patient survival may vary among subregions defined by CD3 cell count and PD-L1 expression levels. With two biomarkers (PD-L1expression and CD3 count) and ten baseline covariates, six survival models were trained, including 4 machine learning models. Applying 5 repetitions of $3\times 3$-fold nested cross-validation, the random survival forest (RSF) method yielded a C-index of 0.84, resulting in the best predictive performance for overall survival, Table 2. Traditional survival models, i.e. Cox proportional hazard regression and survival regression yielded similar predictive accuracy, C-index of 0.70 and 0.71, respectively. Among six predictive models, the deep learning model, DeepSurv, yielded the worst performance with C-index of 0.60. Detail descriptions of hyperparameter tuning for each model are provided in the Supplemental Materials, Table S2.

**Table 2:** Model predictive performance.

Model	C-index (overall survival)	C-index (50% survival at 5-year)	C-index (70% survival at 5-year)
Cox-PH	0.70	0.60	0.64
SR	0.71	0.59	0.65
CoxBoost	0.70	0.60	0.63
RSF	0.84	0.73	0.72
ORSF	0.73	0.63	0.67
DeepSurv	0.60	0.57	0.58

**Figure 1:**
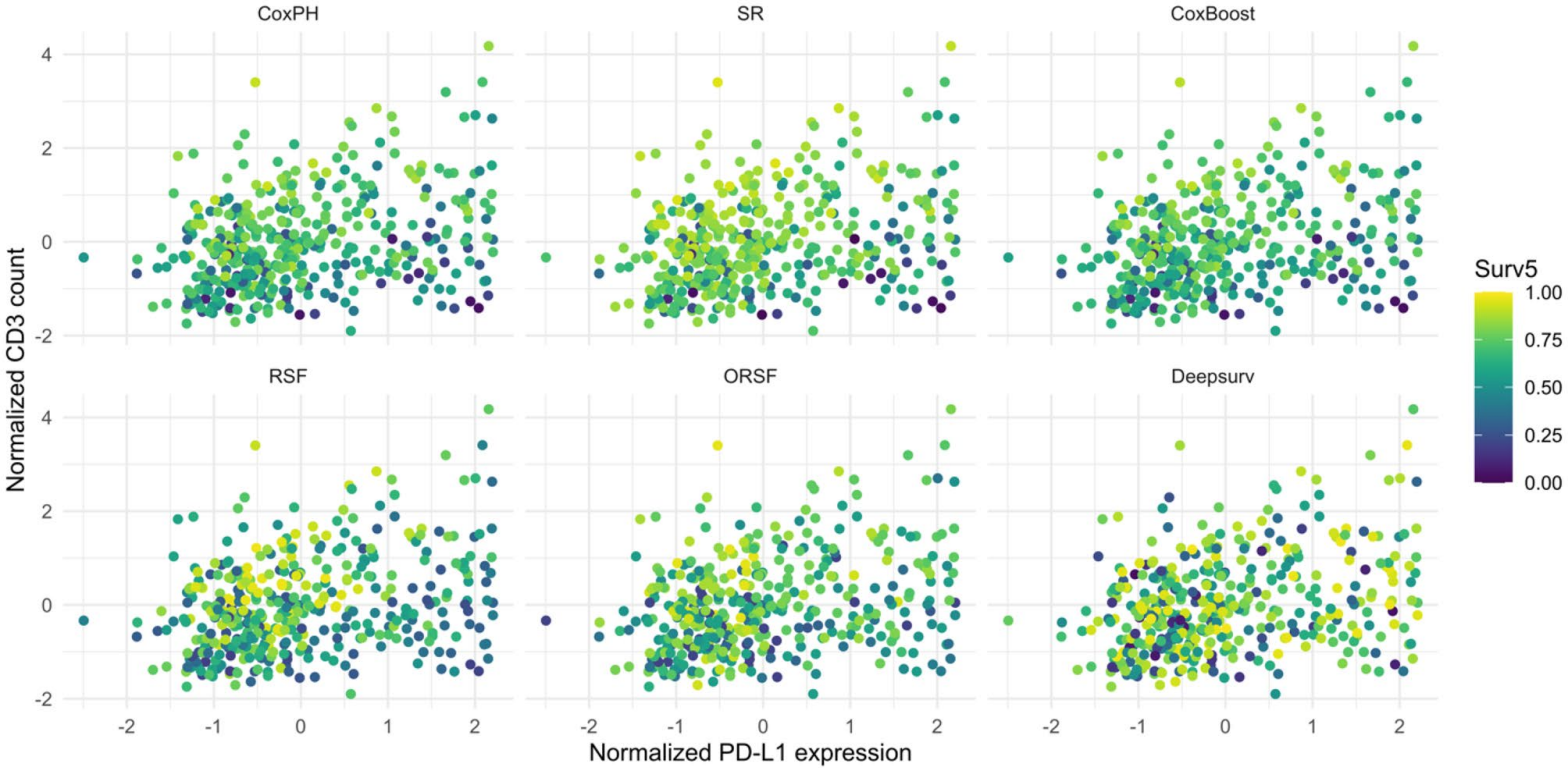
The gradient of predicted 5-year survival probabilities for observed NSCLS patients.

Based on well-tuned predictive models, the 5-year survival probability can be predicted for each observed NSCLC patient. Scatterplots of normalized PD-L1 expression and normalized CD3 are shown in Fig. 1, with color used to depict 5-year survival predictions estimated from each model. Each point on the grid of Fig. 1 represents an observed NSCLC patient profile. The predicted survival probabilities are functions of PD-L1 expression, CD3 count, and ten baseline covariates, Table 1. Patients with high CD3 cell count and low PD-L1 expression have higher survival probability at 5-year compared to other patients on the grid, and patients with high PD-L1 expression have lower survival probability, Fig. 1. This pattern can be better observed in survival regression (SR) and random survival forests (RSF). Due to the low predictive accuracy (C-index = 0.60), the deep learning model, DeepSurv, lacks smoothness, yielding noisy predictions over the grid.

### Subregion pattern

**Figure 2:**
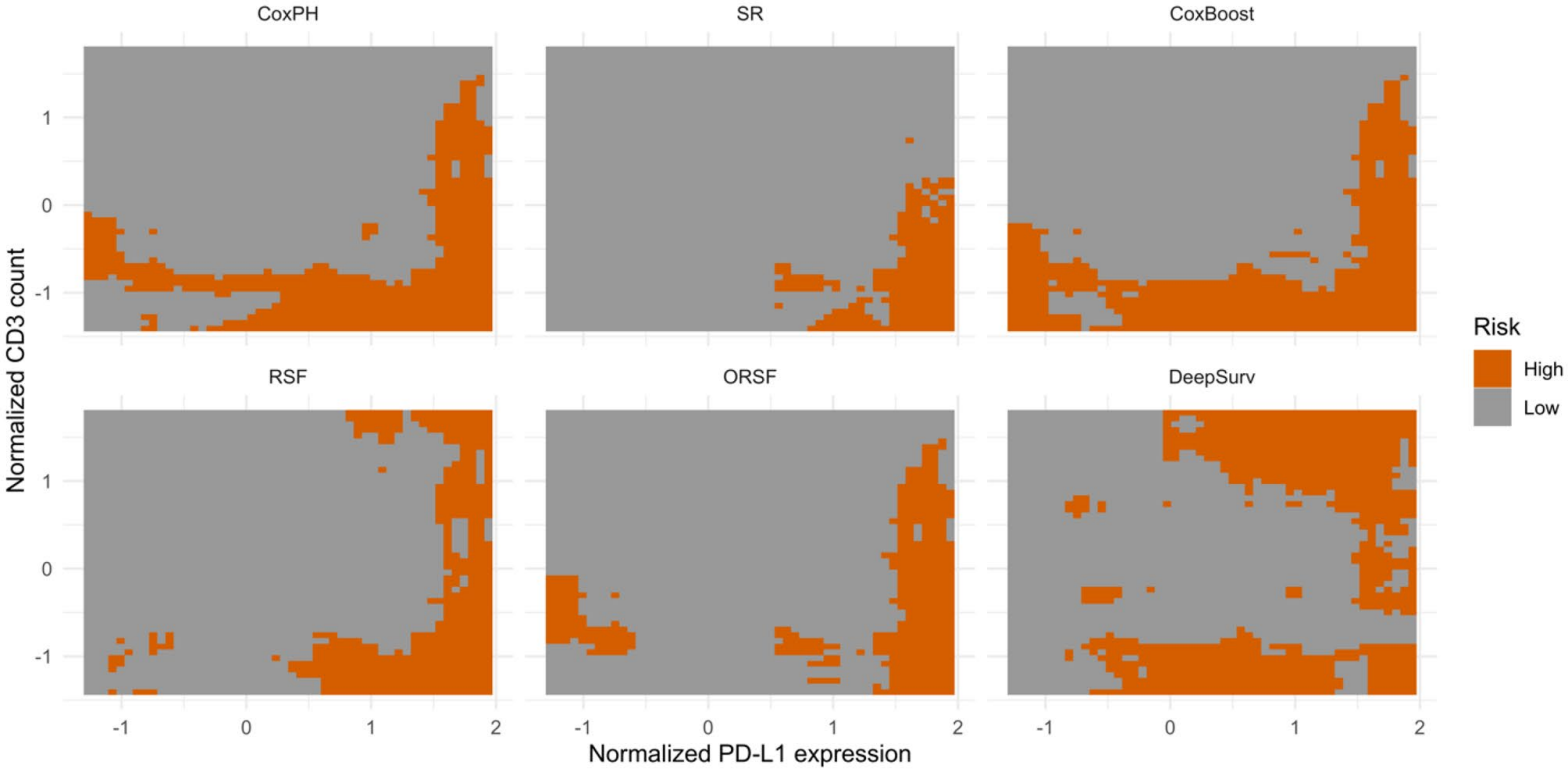
Risk regions over PD-L1 expression and CD3 cell count-defined biomarker space. Regions with high-risk are defined by grid points with 5-year survival probability not greater than 70%. Regions with low-risk are those grid points for which survival probability is greater than 70% at year 5.

To further explore the biomarker subregions, we simulated all possible synthetic patient profiles over the grid (e.g. see “Methods” section). With the 5th and 95th quantiles of the observed values, a 50 $\times 50$ grid of normalized CD3 count and PD-L1 expression percentages with ranges in [− 1.41, 1.78] and [− 1.27, 1.94] is constructed, Fig. 2. Each grid point indicates a synthetic patient profile. For each grid point, a complete patient profile is simulated by imputing clinical prognostic covariates. Consequently, we obtain a total of 2500 synthetic patient profiles over the biomarker region of interest (the CD3 cell count and PD-L1 expression-defined biomarker space). Five-year survival probabilities are predicted for each synthetic patient profile for each model. Prediction gradients over the CD3 cell count and PD-L1 expression-defined biomarker space is shown in the Supplemental Material, Fig. S1.

**Figure 3:**
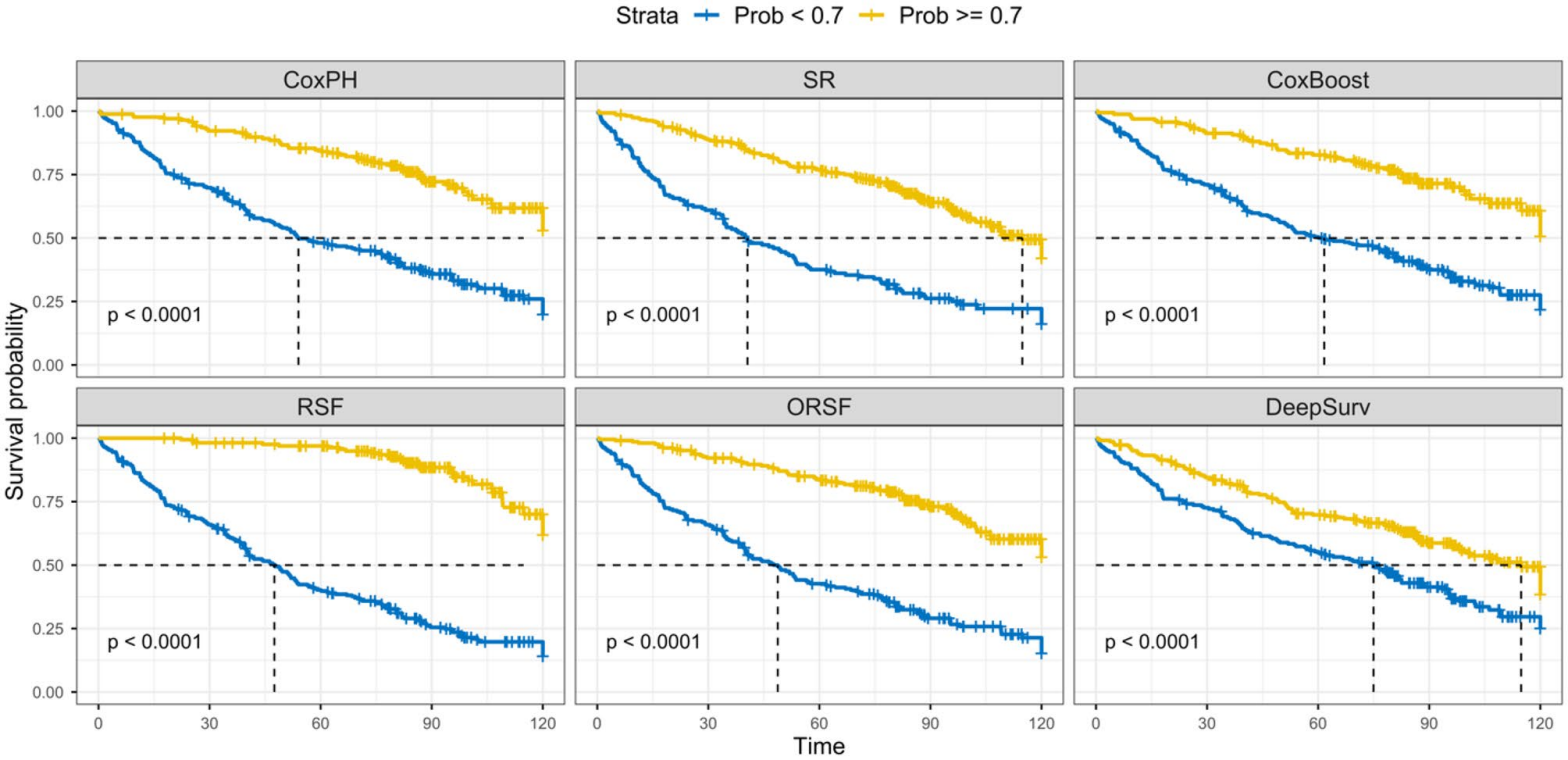
Survival curves comparing high versus low-risk subtypes delineated by a threshold of 70% applied to predicted 5-year survival probability.

We explored prognostic subtypes by applying thresholds to five-year survival predictions, yielding high-risk and low-risk subregions. Figure 2 depicts subtypes resulting from a threshold of 70%. C-indices and survival curves of observed samples are compared between high- versus low-risk subtypes in Table 2 and Fig. 3, respectively. Among implemented models, RSF demonstrated the best discrimination of high- versus low-risk subtypes resulting in a C-index of 0.72, Table 2. All models estimate low 5-year survival expectation for the region with the lowest CD3 cell count and highest PD-L1 expression, i.e. the bottom-right corner of the grid, which is consistent with the previous studies^10,21^.The subregion with high PD-L1 expression also shows relatively low probability of survival, the right region of the grid in Fig. 2, which confirms the conclusion that a high level PD-L1 expression in resected tumor tissue is associated with worse prognosis for NSCLC patients^11,16^. Applying an additional threshold at 50%, the high-risk group in Fig. 2 is divided into high- and medium risk regions, Fig. S2. Owing to the limitations of selecting a single best model, Fig. S3 presents high versus low-risk subtypes delineated using all models' predictions, see details in the Supplemental Materials. The survival curves comparing high versus low risk subtypes delineated by a threshold of 50% to predicted 5-year survival probability is shown in Fig. S4.

## Discussion

Patient heterogeneity is intrinsic to cancer prognosis and pivotal to patient management and selection for clinical trial participation. The characterization of heterogeneity from data is multifactorial, requiring models that accommodate the potential for complex interactions among determinants^23,24^. With the emergence of immune checkpoint inhibitors in recent years, investigators have endeavored to define patient subgroups from biomarkers that describe the extent to which intrinsic induction has occurred within the tumor microenvironment. While authors have proposed techniques for patient subgroup identification from biomarker studies, few have provided solutions for delineating patient subtypes from biomarker subregions that are adjusted for clinical prognosis.

Machine learning models have been widely used in biomedical research^25,26^. For NSCLC patients, Sun et al. investigated the effect of machine learning methods on predicting overall survival (OS) from imaging using radiomic features^27^. This study applied machine learning models to predict the overall survival of 423 surgically resected NSCLC patients. The random survival forest (RSF) demonstrated the best predictive accuracy for overall survival, yielding a concordance index of 0.84. More conventional regression-based models yield concordance indices below 0.71. The DeepSurv model performed worst for overall survival prediction with a C-index of 0.60. The outperformance of RSF algorithm may result from automatic incorporation of variable interactions with nodes split to maximize survival differences. On the other hand, partially due to the small sample size (cancer biomarker studies often do not exceed 500 patients) and the limited number of predictors, the performance of deep learning survival algorithms may require more inputs to outperform RSF.

The resultant predictive models are used for the subsequent subregion pattern analysis. Two biomarkers from TME, the PD-L1 expression and CD3 cell count, were considered for subtype identification. Synthetic patient profiles were simulated from the observed biomarkers and ten clinical prognostic covariates to extend the inference to all plausible regions of the biomarker space. Subregions of biomarker space are identified with survival gradients estimated by predictive models. Such an approach yields an adjusted estimation of biomarker risk gradients, which can be used for subsequent population-level thresholding and subtype identification. It avoids the drawbacks of conventional biomarker thresholding, such as failing to adjust the interdependent patient characteristics or estimate nonlinear biomarker boundaries.

Five-year survival prediction defined low-risk versus high-risk subregions of the biomarker space. The best performing single survival model (random survival forest) estimated an L-shape pattern of prognosis for which very high expression levels of PD-L1 (standardized values > 1.5) reflected poor prognosis for all levels of T-cell CD3 positivity. Low levels of T-cell CD3 positivity, however, were only considered high-risk when combined with PD-L1 expression levels that exceeded standardized values of 0.5 (RSF in Fig. 2; p-value < 0.00001). From these results we conclude that the extent of immunosuppression is the stronger determinant of prognosis for the definitive surgical population of NSCLC. While different models may generate different biomarker thresholds, to overcome the issues of model selection for biomarker threshold estimation, we combined the results among several models to obtain conservative and aggressive thresholds in this study, see details in Supplemental Materials. Both thresholds identified the worst prognostic subtype as defined by patients with low CD3 counts and high PD-L1 expression. We believe that CD3 counts and PD-L1 expression can be used to interrogate the extent to which a patient’s TME reflects an immunosuppressive state versus immune active state. By recognizing tumor-specific antigens and mediating cytotoxic responses, higher concentrations of CD3-positive T-cells reflect more innate immune activity against malignant cells. Increased anti-tumor activity induced by T-cells controls local growth and suppresses distant migration. By way of contrast, overexpression of PD-L1 on tumor cells facilitates engagement with the PD-1 receptor on T-cells, which impairs the effectiveness of the innate antitumor immune response. Therefore, a TME that expresses high levels of PD-L1 evades immune surveillance, reflecting immunosuppressive states, which promotes tumor growth, invasion, and metastasis.

While this trend is consistent with the previous studies^10,21^, the resultant biomarker subtypes identified in this study are more refined. Unlike the beforementioned two studies, where the TME subtypes are identified with predefined thresholds derived by clinical knowledge or optimal cutoffs search to maximize association with OS, this study applied both linear and nonlinear models to estimate the risk gradient for threshold estimation with additional adjustment of baseline covariates. Based on optimal cutoff search without adjustment for clinical prognosis, Tang et al. partitioned the patients into four disjoint quadrants with cut-off points of 1910 cells/mm^2^ for CD3 count and 2% for PD-L1 expression percentage^10^. In this study, the conservative thresholds identified a rectangle subregion of high risk with cutoffs of 1948 cells/mm^2^ for CD3 count and 23% for PD-L1 expression percentage. The aggressive thresholds (1683 cells/mm^2^ for CD3 count and 31% for PD-L1 expression percentage) identified that patients with either low CD3 or high PD-L1 experienced a higher death rate, yielding an L-shaped pattern of prognosis.

This study demonstrated the potential for machine learning to delineate prognostic biomarker subtypes from survival endpoints. A few limitations require consideration. This study was conducted based on data from a single institution without external validation. Although adjuvant therapy is crucial for predicting cancer patient outcomes, due to limited detailed information, this study only considered whether the patient received adjuvant therapy as a binary variable in the predictive model. For patient profiling, only two risk groups are considered. Moreover, risk stratification is based on subjective thresholds for 5-year survival of 70% and 50%, respectively. The biomarker subregions identified is only validated in the available 423 NSCLC patients in this study. Further study is required to provide external validation^28^. Finally, machine learning models use predictions to estimate patterns. Formal statistical hypothesis testing for subtype identification cannot be derived from machine learning models, as they lack asymptotic theory to calculate p-value distributions.

## Materials and methods

### Patients

Surgical resection is the standard treatment for early stage NSCLC patients^29^. The majority (56%) of stage I and II NSCLC are treated with different levels of surgical resection^8^. In this study, pathologically confirmed NSCLC patients who have received surgical resection, diagnosis of non-metastatic disease, and lack of induction of therapy are included. The access date for the research purpose of this data was 09/02/2017. The data used in this study is fully anonymized and investigators do not have the information that could identify individual participants during or after data collection. As part of standard of care, all patients were pretreated with staging chest CT with contrast agent. And adequate surgical pathology specimens were collected from all patients for immunohistochemistry (IHC) staining. Two biomarkers of tumor microenvironment are measured, i.e. percentage of PD-L1 expression and CD3 cell count. An additional ten baseline patient characteristic were collected including: age at surgery, sex, race, smoking status, T status, N status, disease stage, diagnosis of adenocarcinoma and squamous, treatment with adjuvant therapy, and lesion size. The primary outcome is the overall survival. All methods were conducted following the applicable guidelines and regulations. The Institutional Review Board at MD Anderson Cancer Center approved all analyses, and due to the study's retrospective nature, the need for informed consent was waived by Institutional Review Board at MD Anderson Cancer Center.

### IHC analysis and biomarker expression

The IHC analysis used four-micron-thick sequential histologic tumor sections, which were obtained from a representative formalin-fixed, paraffin-embedded tumor block. An automated staining system (BOND-MAX; Leica Biosystems, Nussloch, GmbH) with antibodies against CD3 (T-cell lymphocytes; dilution 1:100; Dako, Carpinteria, CA, USA) and PD-L1 (clone E1L3N, dilution 1:100; Cell Signaling Technology, Beverly, MA, USA) was used for IHC staining. Biomarker expression was detected with a Novocastra Bond Polymer Refine Detection kit (Leica Biosystems) with a diaminobenzidine reaction to detect antibody labeling and hematoxylin counterstaining. And an Aperio AT2 scanner (Leica Biosystems) was then used to scan the slides spanning multiple regions of the tumor. CD3 cell counts were evaluated by a pathologist using Aperio Image Toolbox analysis software (Aperio, Leica Biosystems) and counted as cell density (CD3 cells/mm2 of analyzed tissue). Under the supervision of a pathologist, the percentage of tumor cell PD-L1 expression was quantified with the GENIE histology pattern recognition algorithm (Aperio) for automated identification of the tumor regions using a membrane algorithm.

### Survival analysis and machine Learning methods

The aim of this study is to compare recent innovations in survival analysis to forecast time-to-death for NSCLC patients when combining clinical prognostic factors with biomarkers. Based on the predictive models, we demonstrate how to identify biomarker subtypes of the tumor microenvironment (TME) that are adjusted for clinical factors. This latter aim is mostly neglected in the machine learning literature for biomarker research, for which biomarker studies neglect frameworks for integrating clinical and prognostic factors. Univariate associations between each variable and overall survival was evaluated using Cox proportional hazard model (Cox-PH)^30^, the hazard ratios and P-values are reported. For overall survival prediction, six linear and nonlinear machine learning survival models were implemented with two biomarkers (CD3 cell count and PD-L1 expression of tumor cell) and ten baseline patient characteristics. For linear models, we implemented the traditional survival models including Cox-PH and survival regression model (SR)^31^. For nonlinear machine learning models, we implemented boosting-based, bagging-based, and neural networks-based models, including Cox model by likelihood-based boosting (CoxBoost)^32^, random survival forests (RSF)^33^, Oblique random survival forests (ORSF)^34^, and Cox proportional hazards deep neural network (DeepSurv)^34^. A brief description of each model is given in the section S1 of Supplemental Materials. This study followed established guidelines for developing and reporting machine learning predictive models^26^.

### Model evaluation and calibration

Harrell’s concordance index (c-index)^35^ is used for model calibration and to compare final models for prediction performance. The c-index (which ranges between 0.5 and 1) extends the concept of area under the receiver operating characteristics curve used in classification settings to analyze right-censored time-to-failure endpoints through evaluations of rank-ordered pairs. The statistic synthesizes a model’s predictive utility by comparing the model's estimated predictors and the observed failure time durations among pairs of patients. A pair of patients ($i$ and $j$) are considered “concordant” if patient $j$ lives longer than patient $i$, and the model estimates a higher relative risk of death for patient $i$ when compared to patient $j$. The c-index describes the proportion of concordant pairs. The maximum value of 1 indicates perfect rank-ordering of predictions and observed failure-time durations. A value of 0.5 indicates random rank-ordering with no predictive utility. Model calibration and fitting applied a $3\times 3$-fold nested cross-validation design with five repetitions to tune hyper-parameters using R package mlr3^36^. More details on nested cross-validation can be found in section S2 of Supplemental Materials.

### Data augmentation using synthetic patient profiles

A novel algorithm for data augmentation through synthetic patient profile simulation was applied to extend biomarker prediction beyond the observed sampled data points while also adjusting the gradients for potential correlation among biomarkers and clinical prognostic factors. Synthetic patient profiles that span the entire biomarker regions of interest (ROI) are simulated using the following missing data imputation framework.

1. Generate a grid of patient profiles based on biomarkers, ${D}_{b}=\left\{{X}_{1},{X}_{2},\dots ,{X}_{m}: {X}_{i}\in \left[{\xi }_{i},{\phi }_{i}\right], i=1, 2, \dots ,m\right\},$ where ${\xi }_{i}$ and ${\phi }_{i}$ are the lower and upper bound of $i$-th biomarker on the grid, respectively.
2. At each grid point in the biomarker ROI, apply machine learning imputation technique to impute values of $l- m$ prognostic baseline clinical covariates, which are assumed to be missing, conditional on the observed dataset,${D}_{c}=\left\{{X}_{m+1},{X}_{m+2},\dots ,{X}_{l}\right\}$.
3. Combine synthetic patient profiles from all grid points to generate a data set of synthetic patients with complete values of prognostic covariates that spans the entire biomarker ROI for further analysis, ${D}_{0}=\left\{{X}_{1},{X}_{2},\dots ,{X}_{m},{X}_{m+1},{X}_{m+2},\dots ,{X}_{l}\right\}$.

This algorithm was applied to predict survival from PD-L1 expression and CD3 cell count, i.e., $m=2$. For data analysis CD3 cell count was normalized with Z-score standardization; the percentage of PD-L1 expression was transformed by Z-score standardization on the log-transformed scale. Based on the range of normalized biomarker measurements, a 50 $\times 50$ grid of PD-L1 expression and CD3 cell count was generated. The prognostic baseline covariates were imputed at each grid point in step 2 by the randomForest method using the Missforest^37^ package in R. This accounts for interdependence among biomarkers and clinical prognostic factors, an assumption that is often ignored in practice. Lacking statistical methods for threshold selection that enable adjustment for covariates, investigators often select thresholds on the basis of the biomarkers alone. One should note that this approach requires that the sampled patient population is balanced for all prognostic factors at all regions of the biomarker domain. This is unlikely in the absence of randomized study. Failing to acknowledge correlation between clinical prognosis and TME biomarkers will result in thresholds that misattribute known clinical prognostic trends to biomarkers.

### Survival prediction and subtype identification

Using the synthetic patient profiles generated for each grid point of the biomarker ROI, overall survival was predicted for each of the six models at each grid point. The resultant five-year survival probability gradient was visualized for each model over the biomarker ROI grid. High versus low-risk subtypes were defined for each model by applying thresholds over the probability gradients to five-year survival. The predictive utility of the resultant subtypes is compared between models using the c-index.

### Supplementary Information

Supplementary Information.

## Data Availability

The data is proprietary to MD Anderson Cancer Center and can be shared only with a material transfer agreement. For data access, please contact Dr. Eugene J. Koay (EKoay@mdanderson.org).

## References

[1] Howlader N (2020). SEER Cancer Statistics Review, 1975–2017.

[2] Liao G (2021). Prognostic role of soluble programmed death ligand 1 in non-small cell lung cancer: A systematic review and meta-analysis. Front. Oncol..

[3] Tubin S, Khan MK, Gupta S, Jeremic B (2021). Biology of NSCLC: Interplay between cancer cells, radiation and tumor immune microenvironment. Cancers.

[4] Barta JA, Powell CA, Wisnivesky JP (2019). Global epidemiology of lung cancer. Ann. Glob. Health.

[5] Howlader N (2020). The effect of advances in lung-cancer treatment on population mortality. N. Engl. J. Med..

[6] Siegel RL, Miller KD, Jemal A (2019). Cancer statistics, 2019. CA Cancer J. Clin..

[7] Varela G, Thomas PA (2014). Surgical management of advanced non-small cell lung cancer. J. Thorac. Dis..

[8] Miller KD (2019). Cancer treatment and survivorship statistics, 2019. CA Cancer J. Clin..

[9] Goldstraw P (2011). Non-small-cell lung cancer. The Lancet.

[10] Tang C (2018). Development of an immune-pathology informed radiomics model for non-small cell lung cancer. Sci. Rep..

[11] Azuma K (2014). Association of PD-L1 overexpression with activating EGFR mutations in surgically resected nonsmall-cell lung cancer. Ann. Oncol..

[12] Meyers D, Bryan P, Banerji S, Morris D (2018). Targeting the PD-1/PD-L1 axis for the treatment of non-small-cell lung cancer. Curr. Oncol..

[13] Garon EB (2015). Pembrolizumab for the treatment of non–small-cell lung cancer. N. Engl. J. Med..

[14] Brahmer JR (2012). Safety and activity of anti–PD-L1 antibody in patients with advanced cancer. N. Engl. J. Med..

[15] Glatzel-Plucinska N (2018). SATB1 level correlates with Ki-67 expression and is a positive prognostic factor in non-small cell lung carcinoma. Anticancer Res..

[16] Pawelczyk K (2019). Role of PD-L1 expression in non-small cell lung cancer and their prognostic significance according to clinicopathological factors and diagnostic markers. Int. J. Mol. Sci..

[17] Shimoji M (2016). Clinical and pathologic features of lung cancer expressing programmed cell death ligand 1 (PD-L1). Lung Cancer.

[18] Sun J-M (2016). Prognostic significance of PD-L1 in patients with non–small cell lung cancer: A large cohort study of surgically resected cases. J. Thorac. Oncol..

[19] Zhou C (2017). PD-L1 expression as poor prognostic factor in patients with non-squamous non-small cell lung cancer. Oncotarget.

[20] Cooper WA (2015). PD-L1 expression is a favorable prognostic factor in early stage non-small cell carcinoma. Lung Cancer.

[21] Teng MW, Ngiow SF, Ribas A, Smyth MJ (2015). Classifying cancers based on T-cell infiltration and PD-L1. Cancer Res..

[22] Guo W, Ji Y, Catenacci DV (2017). A subgroup cluster-based Bayesian adaptive design for precision medicine. Biometrics.

[23] Fisher R, Pusztai L, Swanton C (2013). Cancer heterogeneity: Implications for targeted therapeutics. Br. J. Cancer.

[24] Dagogo-Jack I, Shaw AT (2018). Tumour heterogeneity and resistance to cancer therapies. Nat. Rev. Clin. Oncol..

[25] Yu D (2020). Machine learning prediction of the adverse outcome for nontraumatic subarachnoid hemorrhage patients. Ann. Clin. Transl. Neurol..

[26] Luo W (2016). Guidelines for developing and reporting machine learning predictive models in biomedical research: A multidisciplinary view. J. Med. Internet Res..

[27] Sun W, Jiang M, Dang J, Chang P, Yin F-F (2018). Effect of machine learning methods on predicting NSCLC overall survival time based on Radiomics analysis. Radiat. Oncol..

[28] Ou F-S, Michiels S, Shyr Y, Adjei AA, Oberg AL (2021). Biomarker discovery and validation: Statistical considerations. J. Thorac. Oncol..

[29] Heiden BT (2021). Analysis of delayed surgical treatment and oncologic outcomes in clinical stage I non–small cell lung cancer. JAMA Netw. Open.

[30] Andersen PK, Gill RD (1982). Cox's regression model for counting processes: A large sample study. Ann. Stat..

[31] Kalbfleisch JD, Prentice RL (2011). The Statistical Analysis of Failure Time Data.

[32] Binder H, Allignol A, Schumacher M, Beyersmann J (2009). Boosting for high-dimensional time-to-event data with competing risks. Bioinformatics.

[33] Ishwaran H, Kogalur UB, Blackstone EH, Lauer MS (2008). Random survival forests. Ann. Appl. Stat..

[34] Jaeger BC (2019). Oblique random survival forests. Ann. Appl. Stat..

[35] Harrell FE, Califf RM, Pryor DB, Lee KL, Rosati RA (1982). Evaluating the yield of medical tests. Jama.

[36] Lang M (2019). mlr3: A modern object-oriented machine learning framework in R. J. Open Source Softw..

[37] Stekhoven, D. J. & Stekhoven, M. D. J. *Package ‘missForest’. R package version* **1** (2013).

