# Supplementary material for: Machine learning identifies prognostic subtypes of the tumor microenvironment of NSCLC
Source: Sci Rep. 2024 Jul 1;14:15004. doi: 10.1038/s41598-024-64977-7 (PMC11217297; doi:10.1038/s41598-024-64977-7)
Supplement: Supplementary file 1 — Supplementary Information. [file 41598_2024_64977_MOESM1_ESM.pdf]

Supplemental Materials

Duo Yu, Michael Kane, Eugene J. Koay, Ignacio I. Wistuba, Brian P. Hobbs

S1. Supplemental Machine Learning Models
S2. Supplemental Model Calibration and Hyperparameter Tuning
S3. Supplemental Ensembled Thresholding Results
S4. Supplemental Figures
S5. Supplemental Tables

S1. Supplemental Machine Learning Models

Given the right-censored survival data, $\{T_i = \min(Y_i, C_i)), \delta_i = I(Y_i \leq C_i), X_i\}, i = 1,2,\cdots,n,$ where Y_i denotes the observed survival time; C_i denotes the right censored time; and X_i denotes the observed covariates, $X_i = (x_{i1}, x_{i2}, \cdots, x_{ip})'$.

Cox proportional hazard model (Cox-PH)

In survival analysis, one of the fundamental quantities is the hazard function, which is defined as

$$h(t) = \lim_{\Delta t \to 0} \frac{P(t \leq T < t + \Delta t | T \geq t)}{\Delta t}.$$

It represents the instantaneous rate of failure at time t, given that the individual has not experience the event until t.

To study the association between the covariates X and the hazard of Y, a Cox proportional hazard model assumes the noninformative censoring, i.e. C_i is independent of Y_i given X_i, $C_i \perp Y_i | X_i$. And the hazard function is modeled as

$$h(t|X) = h_0(t)\, e^{\beta' X},$$

where $h_0(t)$ is the baseline hazard function, and β is the coefficient. The coefficient vector β is estimated by maximizing the partial log-likelihood

$$L(\beta | X, T, \delta) = \sum_{i=1}^{n} \delta_i \beta' X_i - \log\,(\sum_{l \in R_i} e^{\beta' X_l}),$$

where X_i denotes the observed covariate vector of the i-th subject, and R_i is a set of subjects who are at risk at time T_i. The model implies the proportional hazard property, i.e.

$$\frac{h(t|X = x_1)}{h(t|X = x_2)} = \frac{h_0(t) e^{\beta' x_1}}{h_0(t) e^{\beta' x_2}} = e^{\beta'(x_1 - x_2)}.$$

Without loss of generalization, we assume an univariate analysis, then the hazard ratio can be expressed as

$$\frac{h(t|X = x + 1)}{h(t|X = x)} = e^{\beta}.$$

Therefore, the statistical association between covariate X and the time to event can be fully studied through the coefficient β. Due to such straight-forward interpretation property, the Cox proportional hazard model is so far the most popular statistical model in survival analysis of the biomedical problems. The Cox proportional hazard model is also usually called Cox model.[32]

Survival regression model (SR)

Survival regression is a class of full parametric models in survival analysis. It directly models the relationship between survival time and covariates:

$$\log(Y) = \mu + \gamma' X + \sigma W, \qquad W \sim F$$

where F is a known distribution, such as standard extreme value distribution, logistic, normal etc. For example, if the F follows a standard extreme value distribution, it leads the Weibull regression model:

$$\log(Y) = \mu + \gamma' X + \sigma W, \qquad W \sim standard\ extreme\ value\ distribution$$

This model is equivalent to a proportional hazards model for Y with a Weibull baseline hazard, that is,

$$h(y|X) = \alpha \lambda y^{\alpha - 1} \exp(\beta' X),$$

with $\alpha = 1/\sigma$, $\lambda = \exp\,(-\mu/\sigma)$, and $\beta_j = -\gamma_j/\sigma, j = 1,2,\cdots,p$.

Boosted Cox regression (CoxBoost)
Boosting is an iterative machine learning technique that can be applied in various of statistical problems, including classification, regression and survival analysis. [33-35] A boosted Cox regression is a likelihood-based boosting algorithm in the Cox model. The parameters are iteratively estimated through maximizing the following function

$$l(\beta|X,T,\delta,\hat{\beta}) = \sum_{i=1}^{n} \delta_i[\hat{\eta}_i + \beta' X_i - \log(\sum_{l \in R_i} e^{\hat{\eta}_l + \beta' X_l})],$$

where $\hat{\eta} = \hat{\beta}' X$ is the offset term that links the iteratively estimated parameters. [36]

Random survival forests (RSF)
Random survival forests (RSF) is an ensemble tree method for right-censored survival data analysis.[20] It extends the random forests, in which a collection of decision trees are built for classification and regression,[37] to time to event setting.

To adapt the idea of random forests, RSF uses the ensemble mortality as the predicted outcome which comprises both the survival time and censoring information. The expected outcome is defined as the sum of estimated cumulative hazard function (CHF) over the observed time (both censored and uncensored). For a subject i, the expected mortality can be expressed as

$$M_i = E_i\left(\sum_{j=1}^{n} H(T_j|X_i)\right)$$

where X_i is the covariate vector of subject i, T_j is the observed time for subject j, and $H(T_j|X_i)$ denotes the value of CHF at time T_j, conditioning on X_i.[20] Then, the ensemble mortality for subject i is defined as

$$M_{e,i} = \sum_{j=1}^{n} H_e(T_j|X_i)$$

where $H_e(T_j|X_i)$ is the ensemble CHF at time for T_j subject i with covariates X_i, and $H_e(T_j|X_i) \in [0, \infty)$. Therefore, estimating ensemble CHF has been the key step in the RSF algorithm. Specifically, the ensemble CHF at time t is estimated as

$$\hat{H}_e(t|X_i) = \frac{1}{B}\sum_{b=1}^{B} \hat{H}_b(t|X_i)$$

where B denotes the total number of trees; $\hat{H}_b(t|X_i)$ is the estimated CHF in b-th tree. Given an individual survival tree and X_i, subject i will fall into a unique terminal node of the tree. For the b-th tree, assume subject i falls into the terminal node, h. Then, for the b-th tree, the CHF can be estimated with various estimators, including the Nelson–Aalen estimator which can be expressed as

$$\hat{H}_b(t|X_i) = \hat{H}_{b,h}(t) = \sum_{t_{h,l} \le t} \frac{d_{h,l}}{Y_{h,l}}$$

where $d_{h,l}$ and $Y_{h,l}$ are the number of deaths and number of subjects at risk at time $t_{h,l}$ in the terminal node h, respectively.

Following the random forests algorithm, the RSF algorithm can be described as

1) Random generate a boostrap training samples from the original data set.
2) Grow survival tree for the boostrap samples. At each node, random select m variables to split the node into two daughter nodes with the aim of maximizing the survival difference between the daughter nodes. The tree is built until the terminal node has no less than s >0 deaths.
3) Calculate CHF for each tree
4) Repeat 1)-3) B times
5) Calculate the estimated ensemble CHF based on B individual trees.

Oblique random survival forests(ORSF)
Oblique random survival forests (ORSF) is also an ensemble tree-based method for right-censor survival analysis.[21] It is an extension of random survival forests (RSF).[20] It generalizes RSF by using linear combination of variables to recursively partition the training data.

Cox proportional hazards deep neural network (DeepSurv)
DeepSurv is a deep learning method of survival analysis. It generalizes the Cox-PH model by modeling the hazard function as

$$h(t|X) = h_0(t)\, e^{f(x)}$$

where $f(x)$ is output of a deep feed-forward neural network. [38]

S2. Supplemental Model Calibration and Hyperparameter Tuning
Nested cross-validation
Cross-validation is a widely employed technique for both model selection and performance evaluation.[39] Instead of training the model only once on the training data set which may generate over-fitting problem, to reduce the generalization error, cross-validation provides a simple way to reduce the bias by sampling. A k-fold cross-validation randomly split the training data set into k non-overlapping folds; the model is then repeatedly trained k times. In each fold of the cross-validation procedure, one fold of the data is left out for performance evaluation; and the rest of $k-1$ folds are used for training (i.e., parameter estimation with some optimization algorithm). The model performance is evaluated by the averaged value of k performance measures. Based on the k-fold cross-validation, the performance of different types of models can be compared and model selection processes can be conducted.

However, machine learning models commonly comprise one or more hyper-parameters which allow the model to adaptively fit the specific patterns given different data sets. For example, the hyper-parameters of random survival forests (RSF) are number of trees, node size, split rules, number of variables that randomly selected for node splitting, et. al..[20] Usually, these hyper-parameters are tuned by grid-search or random search with cross-validation. A nested cross-validation is an approach to conduct hyper-parameters tuning and model selection simultaneously. It nests the cross-validation for hyper-parameters tuning inside the cross-validation for model selection.

Hyperparameter tuning
Given a list of hyperparameters from each model, the optimal set of hyperparameters is tuned with 5 repetitions of 3×3 folds nested cross-validation. The optimal set of hyperparameters is the set

that reaches largest mean C-index of inner training and testing datasets. The final selected hyperparameters are shown in the Table S2.

S3. Supplemental Ensembled Thresholding Results

Owing to the limitations of selecting a single best model, Figure S3 presents high versus low-risk subtypes delineated using all models' predictions. Two scenarios of thresholding are explored as follows.

- Conservative thresholds: the boundaries are defined by the maximum 5-year survival probability among six implemented models; see the top left panel in Figure S3.
- Aggressive thresholds: the boundaries defined by the minimum 5-year survival probability in 6 implemented models; see the bottom left panel in Figure S3.

With a cut-off of 70% for 5-year survival probability, conservative thresholds identify a rectangle-shape of risk region, depicted in the top left panel in Figure 2. This indicates that patients with a high PD-L1 and low CD3 cell count increased death rate. On the other hand, patients with tumors with either low PD-L1 or high CD3 cell counts experience a higher 5-year survival probability. Aggressive thresholds identify a L-shape of the risk region, the bottom left panel in Figure 2. Both thresholding techniques result in statistically significant subtypes, as shown in the right panels of Figure 2.

S4. Supplemental Figures

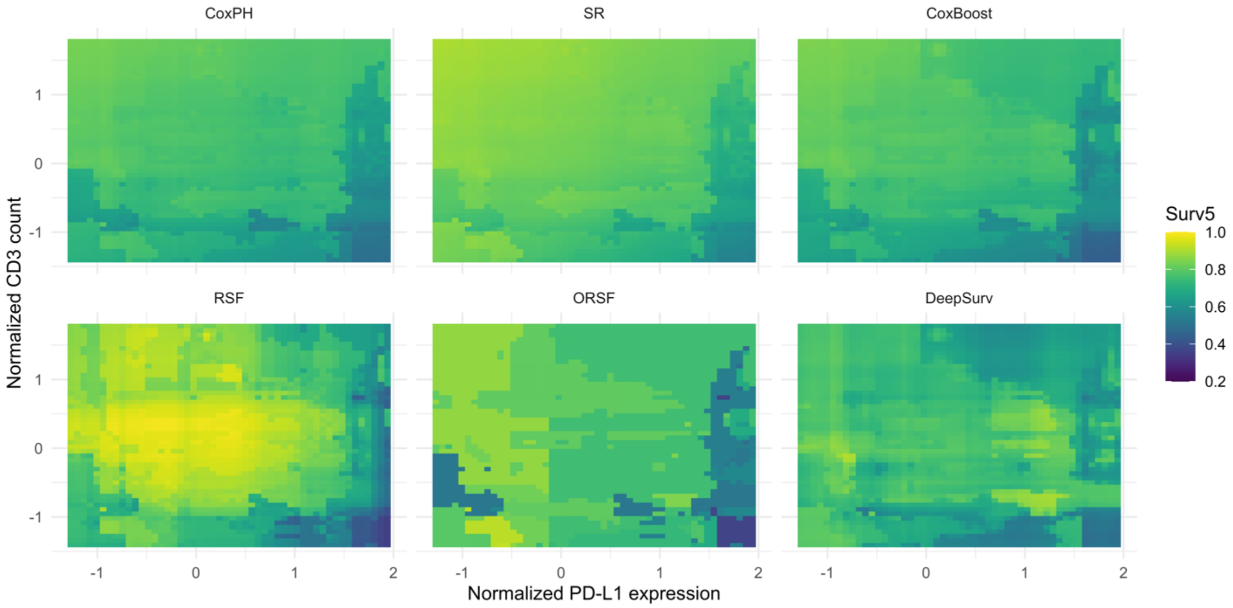

Figure S1. Gradient of the survival probability at 5-year over PD-L1expression and CD3 cell count-defined biomarker space.

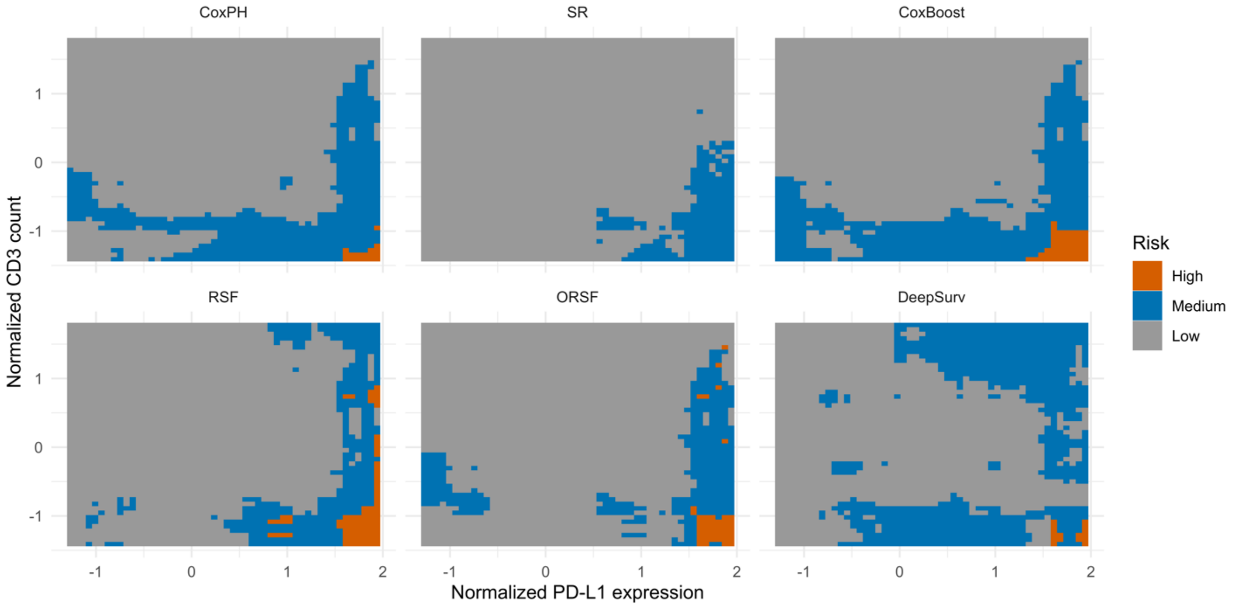

Figure S2. Risk regions over PD-L1expression and CD3 cell count-defined biomarker space. Regions with high-risk are those grid points have survival probability not greater than **50%** at 5-year. Regions with medium-risk are those grid points have survival probability greater than 50%

but not greater than 70% at 5 year. Region with low-risk are those have survival probability greater than 70% at 5-year.

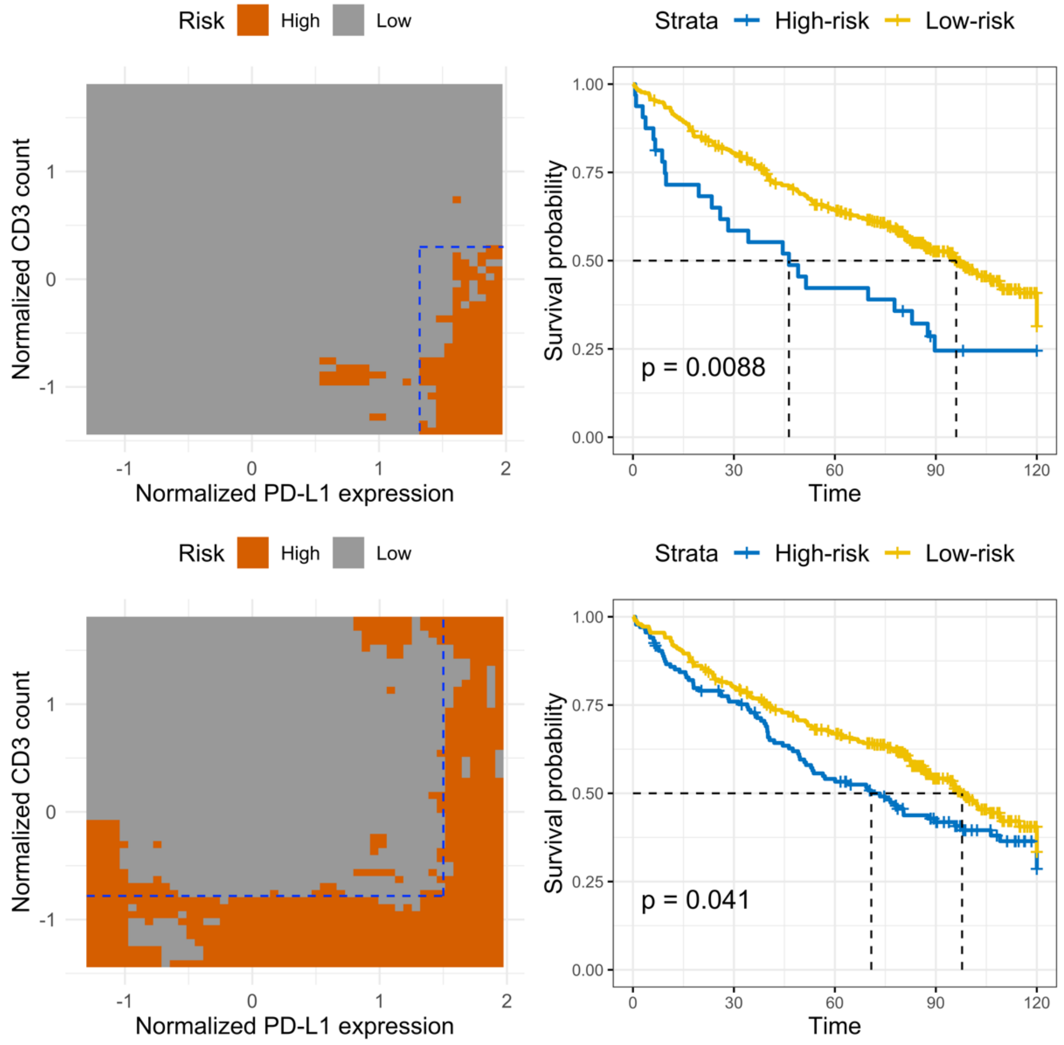

Figure S3. CD3 cell count and PD-L1 expression level thresholding and corresponding risk subregions in biomarker space (left panels) and stratified risk group among the observed samples (right panels). The top left panel is the conservative threshold defined by the maximum 5-year survival probability in six implemented models; the bottom left panel was computed from the aggressive threshold defined by the minimum 5-year survival probability in six implemented models.

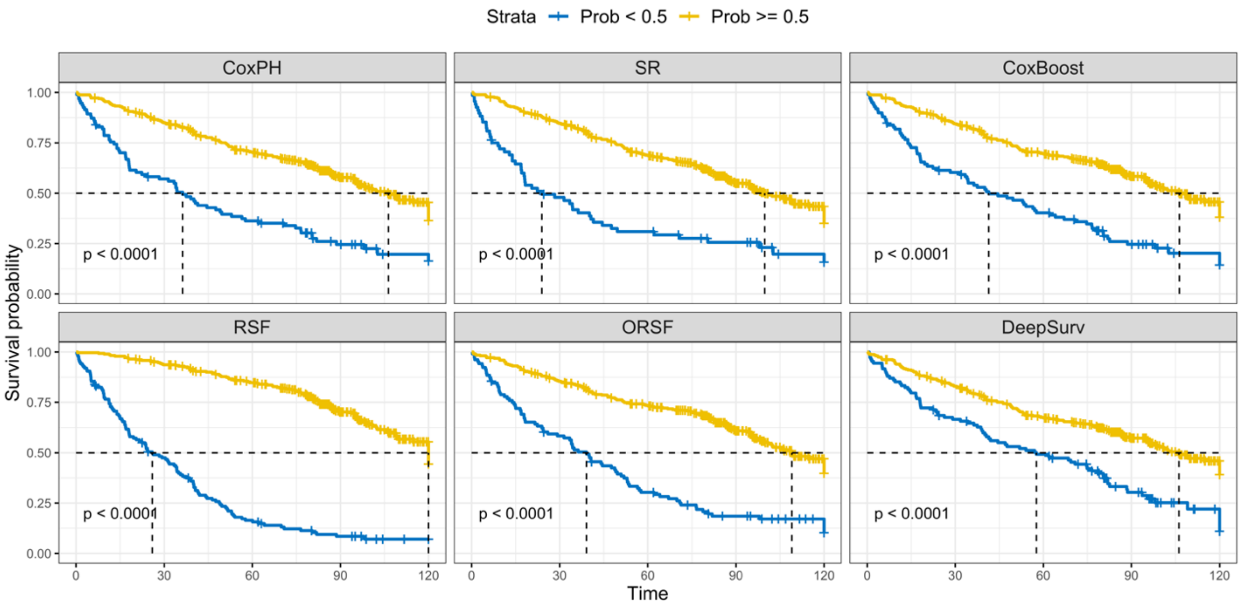

Figure S4. Survival curve based on the cut-off of 50% survival probability at 5-year.

S4. Supplemental Tables

Table S1: Summary of adjuvant therapy types.

Adjuvant therapy types	Number of patients
Chemotherapy alone	103
Radiation alone	19
Chemotherapy + Radiation	18
Others	4

Table S2. Methods parameter tuning and corresponding performances.

method	parameters	Range of parameters	Tuned values
Cox-PH	-	-	-
survival regression	Model form	Accelerated Failure Time (aft), Proportional Hazards (ph), Proportional Odds (po), Tobit (tobit)	ph
	distribution	Gaussian, Weibull, exponential, loglogistic, lognormal	loglogistic
Coxboost	Number of boosting steps	[500,5000]	4174
	Step size	[-2,2]	1.67
	penalty	[0,2]	1.55
RSF	ntree	[500,5000]	4877
	Node size	[1,10]	8
	mtry	[1,10]	9
	splitrule	logrank, bs.gradient, logrankscore	logrank
ORSF	ntree	[100,500]	312
	Leaf node size of observation	[1, 10]	9
	mtry	[0,10]	5
	dfmax	[1,3]	3
	nsplit	[1,20]	19
DeepSurv	Dropout	[0,1]	0.74
	optimizer	adagrad, adam, rmsprop, sgd	sgd
	Weight decay	[0,0.5]	0
	Learning rate	[0,1]	0.69
	alpha	[0,1]	0.37
	Number of nodes	[1,32]	9
